# Farmers’ and millers’ experiences and attitudes towards the production and processing of zinc biofortified wheat in Pakistan: a mixed methods study

**DOI:** 10.3389/fnut.2023.1158156

**Published:** 2023-10-24

**Authors:** Marena Ceballos-Rasgado, Subhan Ajmal, Usman Mahboob, E. Louise Ander, Munir Zia, Victoria Hall Moran, Edward J. M. Joy, Mukhtiar Zaman, Heather Ohly, Nicola M. Lowe

**Affiliations:** ^1^Centre for Global Development, University of Central Lancashire, Preston, United Kingdom; ^2^Independent Researcher, Faisalabad, Pakistan; ^3^Institute of Health Professions Education and Research, Khyber Medical University, Peshawar, Pakistan; ^4^Inorganic Geochemistry, Centre for Environmental Geochemistry, British Geological Survey, Nottingham, United Kingdom; ^5^Research & Development Department, Fauji Fertilizer Company Ltd, Rawalpindi, Pakistan; ^6^Department of Population Health, London School of Hygiene & Tropical Medicine, London, United Kingdom; ^7^Department of Pulmonology, Rehman Medical Institute, Peshawar, Khyber Pakhtunkhwa, Pakistan

**Keywords:** biofortification, farmers, millers, staple crops, producers, acceptability, wheat value chain

## Abstract

**Background:**

Zinc biofortified wheat may be a sustainable strategy to increase zinc intake in areas where fortification and dietary diversification are not feasible or are limited by household purchasing power. This convergent mixed methods study aimed to explore the farmers’ and millers’ experiences and attitudes towards the production and processing of zinc biofortified wheat in Pakistan.

**Methods:**

A telephone survey was conducted with farmers (*n* = 418) who were provided with Zincol-2016 biofortified wheat seed for the 2019–2020 growing season, as part of a wheat grain micronutrient mapping study across Punjab Province. The survey explored the farmers’ experiences of growing Zincol-2016 and whether they opted to grow it again in the subsequent season. Semi-structured focus group discussions were undertaken in a separate group of farmers in Khyber Pakhtunkhwa (KP) province (*n* = 12) who grew Zincol-2016 for the BiZiFED2 RCT. Millers were also interviewed in KP, both those who had processed Zincol-2016 for the trial (*n* = 12) and those who had no experience of processing biofortified wheat (*n* = 12). Survey data were analyzed using descriptive statistics and transcripts of focus groups were analyzed using thematic analysis.

**Results:**

Nearly half of farmers who responded to the survey (47%) re-cultivated Zincol-2016 in the following season. The drivers for Zincol-2016 re-cultivation were seed availability (100%), grain yield and growth resistance (98%), quality of the flour from the previous harvest (97%) and nutritional benefit (94.5%). Discussions with farmers suggested that the main motivators for potential scale-up of biofortified wheat were the perceived quality of the grain, wheat, and flour. Millers saw it as an opportunity to expand their business. Farmers and millers valued the health benefits of the wheat. Challenges for scale-up include the need of additional support to produce it, unfamiliarity with the biofortification process, production costs, and external threats to the supply chain.

**Conclusion:**

Farmers and millers showed a strong implicit preference for Zincol-2016 over alternative varieties. Crop performance and product yield were the most cited motivators for growing Zincol-2016. Farmers and millers are willing to produce and process biofortified wheat if financial and educational support is provided.

## Introduction

Mild-to-moderate zinc deficiency may lead to growth faltering in children, impaired immune function, and altered integrity and function of the gastrointestinal tract (1). Zinc deficiency remains a serious public health problem, particularly in low-middle income countries. In Pakistan, 22.1% of women of reproductive age and 18.6% of children under 5 years of age are zinc-deficient (2). In rural areas of Pakistan, where diet diversity is low, we recently reported that 68.8% of adolescent girls were zinc deficient (3).

Leading public health strategies to address zinc deficiency include supplementation, fortification and increasing dietary diversity. However, the sustainability of these strategies can be challenging in remote impoverished rural areas where access to affordable diverse diets, supplementation interventions and centrally fortified food products are limited (4). Biofortification is a process by which the density of vitamins and minerals in the edible component of a crop are increased through conventional plant breeding, transgenic techniques, agronomic practices, or a combination of these (5). Agronomic biofortification refers to the addition of nutrient rich fertilizer which can be applied through foliar (fertilization to plant leaves and steams) or basal (pre-plating fertilization) methods. Recent evidence has demonstrated that that foliar application of zinc can increase the zinc concentration and bioavailability in wheat grain and flour (6).

There is increasing evidence that consumption of biofortified foods improve micronutrient status (7, 8). Biofortification strategies consider the specific nutrient needs of the population and the staple foods of the region so that reach, and affordability are maximized (9). If these criteria are met, biofortification of staple crops presents a potential long-term cost-effective and self-sustaining strategy for increasing dietary micronutrient intake (10), in contrast to supplementation and commercial fortification programs which incur higher ongoing costs to sustain them (5).

The success of biofortification strategies to improve micronutrient status on a population scale depends not only on the evidence of a positive impact on relevant health outcomes, but also on high rates of adoption and consumption by the producers and intended beneficiaries (11, 12). Systematic reviews have provided evidence that there is sensory acceptability (11) and a willingness to pay (13) for biofortified crops among consumers. However few studies have explored the views of wheat farmers (14–17), and none to our knowledge have sought the opinions of the millers who process the resulting grain. Studies in Nigeria and Uganda found that farmers have limited knowledge of biofortification (14) or the benefits of fertilizer application (17) and that an increased awareness of the benefits of and positive perceptions towards the biofortified crop were strong determinants of its adoption (14). Two studies investigated farmers’ opinions on the hypothetical introduction of genetically modified maize in Mexico (15) and biofortified pearl millet in India (15). Both studies showed significant heterogeneity among farmers’ views, dependent on their location (related to soil quality, yield, their involvement in local labor markets), age group, and whether they produce mainly for household consumption or market sale. This heterogeneity among farmers’ views towards biofortified crops demonstrates the importance of tailoring biofortification strategies to local needs by considering the views and expectations of local producers.

In 2016 a new variety of zinc biofortified wheat (Zincol-2016) was released by HarvestPlus in partnership with the Research & Development Institutions of Pakistan. As wheat is the main staple food and cultivated on the largest acreages in Pakistan (18), this biofortified crop is a promising approach to improve zinc intake on a population level, especially when combined with zinc fertilizers (6, 19). The BiZiFED2 (Biofortification with Zinc and Iron for Eliminating Deficiency, BBSRC Global Challenges Research Fund, Grant Number BB/S013989/1) trial was established to investigate the potential of biofortification as a strategy to reduce zinc and iron deficiencies in Pakistan. At the start of the study, Zincol-2016 was the only variety of selectively bred zinc biofortified wheat available in Pakistan. The primary objective was to examine the effects of consuming zinc-biofortified wheat flour on the zinc status of adolescent girls aged 10–16 years. We have found that consumption of zinc-biofortified wheat grown with zinc fertilizers has a positive impact on total dietary zinc intake (3, 20) and is perceived positively among consumers (21). Here we present the findings of a convergent mixed-methods study, to explore the views and experiences of the famers and millers of zinc biofortified wheat and flour to inform future programs seeking to scale-up zinc biofortified wheat in Pakistan.

## Materials and methods

This study is part of the BiZiFED2 project (Biofortification with Zinc and Iron for Eliminating Deficiency) (3). One of the main objectives of this research was to improve understanding of the socio-cultural factors and market systems that affect the sustainable uptake of biofortified wheat in Pakistan. To achieve this objective, a mixed method study was undertaken to explore the views and perspectives of farmers, millers and community members to identify what factors influence decisions around their acceptance of biofortified wheat.

### Study procedures

#### Study design

The study used a mixed methods convergent parallel design in which two independent strands of complementary quantitative and qualitative data were collected and analyzed independently and merged in the integration phase (22). This approach was chosen because it would allow us to triangulate our results and obtain a multidimensional understanding that would have not been available through separate qualitative or quantitative approaches. Data were collected using a survey of farmers recruited to cultivate Zincol-2016 the prior growing season as part of the larger BiZiFED2 effectiveness trial (23). Focus group discussions (FGDs) were carried out with farmers and millers who produced zinc biofortified wheat and flour for the BiZiFED2 trial. One additional FGD was conducted with millers who had no experience of milling biofortified wheat.

#### Survey: recruitment and implementation

In 2019, 686 farmers across the Punjab region were recruited to participate in the BiZiFED trial. They were provided (free of charge) with 25 kg Zincol-2016 wheat seed sufficient for 0.5 acre (~0.2 hectare) using standard broadcast sowing practices. Farmers who had granted their permission to be followed up during the initial recruitment period were contacted to take part in a survey. Up to three attempts were made to reach each farmer by telephone. Upon contact, the nature of the study was explained to each potential participant and consent was reconfirmed. Farmers were provided with the contact details of the researchers and encouraged to ask any questions and seek clarification from the research time if required. The survey was conducted by an experienced agronomy extension worker (SA) who was fluent in the local language and trained in the skills required to collect data for this project by telephone, using KoboCollect software (24). The survey was conducted by telephone to maximize response rate and minimize risks of exposure to COVID-19 for all concerned.

The survey was designed to capture the extent of biofortified wheat cultivation, the farmers’ experiences of growing Zincol-2016 in the growing season 2019–2020, and whether they had continued to grow Zincol-2016 in the 2020–2021 growing season. The full suite of questions is provided in Supplementary file 1. Survey data were collected between 10th February and 1st July 2021. Participant responses were captured in KoboCollect software (24) using a handheld tablet and the survey took between 4 and 15 min to complete depending on the number of questions responded to (i.e., farmers who had sown Zincol-2016 in the second growing season were asked more questions than farmers who did not). The survey responses were checked by the research team after the first 10 surveys were conducted to ensure that the survey was working as intended for the participants and the researchers. This revealed that more farmers than expected were growing a second biofortified wheat variety “Akbar-19”, that was released in Pakistan in 2019. Therefore, one additional question was added to the survey to establish the acreage given to growing Akbar-19 on farms cultivating this variety. This enabled us to more accurately capture the production acreage given to biofortified wheat on each farm, as reported by the farmer in the remaining interviews.

#### Focus groups: recruitment and implementation

Four FGDs were planned of which two were with farmers and two with millers. The farmers were recruited from a total pool of 59 tenant farmers cultivating land belonging to two landlords in the Peshawar area. Farmers were small scale farmers, relying mostly on manual techniques for sowing and harvesting and who were given resources (Zincol-2016 seed and zinc fertilizer) to grow Zincol-2016 which was then purchased for use in the BiZiFED2 effectiveness trial. The millers were recruited from two mills that were initially chosen to process the wheat for the trial flour. These mills were selected according to the affordability, accessibility, and condition of the machinery. Of these two mills only one (Mill 1) was chosen to grind the Zincol-2016 wheat grain for the trial. The second mill (Mill 2) acted as a potential substitute in case of any mechanical fault or power shortages in the area. Only non-biofortified wheat was processed in Mill 2, which was used at the start of the trial as a source of control flour. For the FGDs, potential participants were eligible if: they were over the age of 18, were employed at the selected farms or mills, and could willingly give informed consent. The selection of the participants for the FGDs was conducted by the BiZiFED2 RCT trial management team who identified individuals who were willing to speak openly.

A total of four FGDs with a duration of between 30 and 60 min were conducted between November and December 2020. The location of the FGDs was selected based on ease of access for the participants and where COVID-19 safety measures could be ensured. Topic guides were used to lead the FGDs (See Supplementary file 1) and were designed to gather information about local farming/milling practices, their views on biofortified wheat and their willingness to continue to use it, and any challenges farmers and millers may have faced during the COVID-19 pandemic. Topic guides were reviewed by all members of the research team and translated into the local language (Pashto). Each FGD was facilitated by two research assistants who were fluent in the local language and received training from experienced qualitative researchers prior to conducting the FDGs. FGDs were audio recorded and transcribed and translated into English by an independent third-party provider in the UK.

### Data analysis

Simple descriptive statistics, such as frequencies and percentages, were used to analyze the survey data, using Microsoft Excel for Microsoft 365 MSO (Version 2,208).

FGD transcripts were imported into NVivo^®^ 12 (QSR International) for analysis. An inductive thematic analysis was conducted to generate themes following the approach by Braun and Clarke (25). This approach involves an iterative process of six phases: familiarization with the data, generating initial codes, searching for themes, reviewing themes, defining, and naming themes, and producing the report. One researcher in the UK (MCR) and one researcher in Pakistan (UM) independently read and re-read the transcripts to familiarize themselves with the data and undertook an initial coding of the dataset. Similarities and dissimilarities in the coding were discussed between the two researchers until a consensus regarding the overarching codes was achieved. One researcher (MCR) collapsed the codes into themes and sub-themes and a coding tree was generated. This coding tree was reviewed by a second (UM) and third researcher (VHM) and checked against the transcripts. Adjustments were made until consensus was achieved. Further coding and reviewing were undertaken by a researcher (MCR) using the final coding tree. Examples of quotations that best exemplified each theme and sub-theme were chosen by one researcher (MCR) and reviewed by a second researcher (VHM).

Following the analysis of the qualitative and quantitative data, results were carefully examined, and parallels and contradictions were identified, interpreted, and integrated into the discussion.

## Results

### Punjab farmers’ survey

The participation rate for the survey was 61%, with 418 of 686 farmers who were initially contacted agreeing to take part. The findings related to: biofortified crop cultivation; drivers of Zincol-2016 second season cultivation; use of the 2020 harvested Zincol-2016 grain; agronomy and training priorities; and pandemic effects.

#### Biofortified crop cultivation

Of all farmers surveyed, 278 (67%) kept some or all their harvested Zincol-2016 grain separated from their usual variety post-harvest, while the remaining 33% mixed it with any other variety. Of the farmers who kept some separate, some 227 (82%) reported storing this seed for growing in the 2020–2021 season. Almost half of the farmers (47%, *n* = 197) reported that they chose to grow Zincol-2016 again in the following season (2020–21). Nearly two thirds (61%, *n* = 253) of all farmers were aware of Akbar-19, a recently released zinc biofortified wheat variety.

#### Agronomy and training priorities

The majority of farmers did not use foliar zinc fertilization on their crops to increase grain zinc (83%, *n* = 191/230).When asked what factors would influence their decision to apply basal or foliar zinc fertilizer, which would typically incur additional expense for the farmer, the most frequent responses were cost of zinc fertilizer (*n* = 138), whether there was sufficient market demand/buyers interest in the biofortified product (*n* = 130), and lack of knowledge on how to apply foliar zinc fertilizer (*n* = 102), while discoloration of leaves (*n* = 25), preference of organic matter (*n* = 26) and access to credit (*n* = 32) were less commonly identified as influencing factors. Most farmers (82%, *n* = 189/230) indicated they would be more likely to use foliar zinc on wheat intended for their own consumption. Most participants (84% *n* = 341/407) indicated that they would appreciate some training on this approach.

#### Drivers of Zincol-2016 second season cultivation

**Figure 1:**
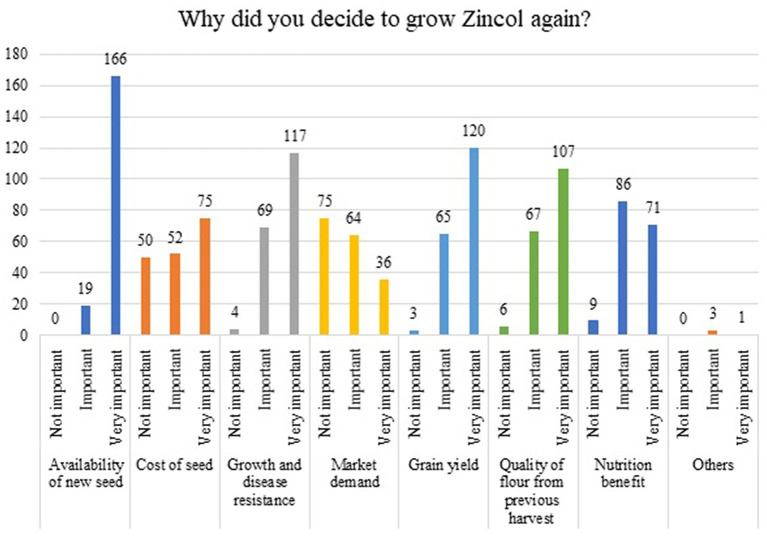
Participant farmers importance attached to selected attributes influencing their decision to grow Zincol-2016 in the follow-on growing season.

The 197 participants who reported growing Zincol-2016 again in the following season were asked to rate how important certain factors were in their decision to grow this variety again. The farmers indicated that the availability of Zincol-2016 seed (*n* = 166/185), growth and disease resistance (*n* = 117/190), grain yield (120/188) and the quality of flour from the previous harvest (107/180) were very important motivators to growing Zincol-2016 again. Nutritional benefit was identified as “important” (*n* = 86/166) or “very important” (*n* = 71/166) by most participants. The cost of seed was rated as “not important” by 28% of those who responded to this option (*n* = 50/177) Figure 1.

#### Use of the 2020 harvested Zincol-2016 grain

**Figure 2:**
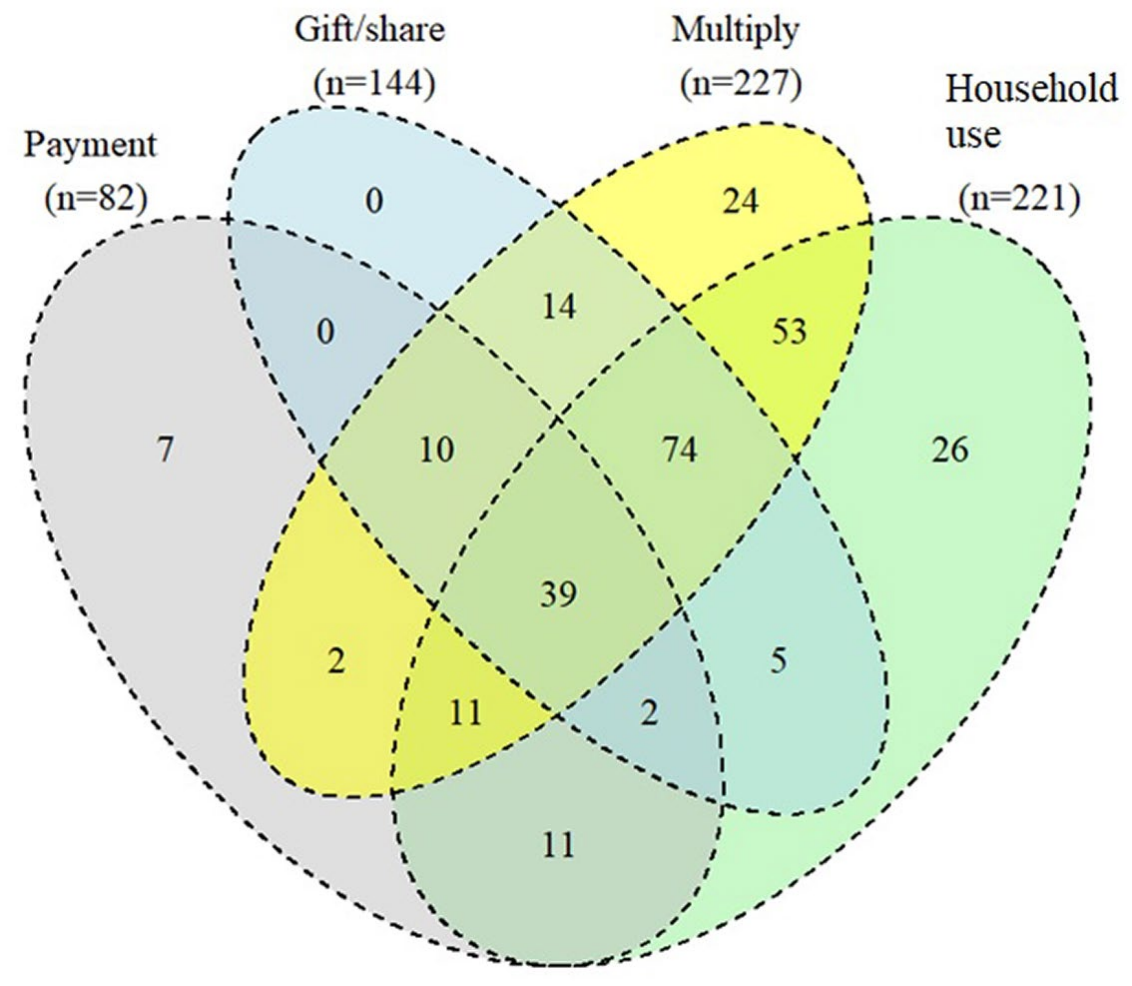
The use of 2020 harvested Zincol-2016 grain reported by participants.

**Figure 3:**
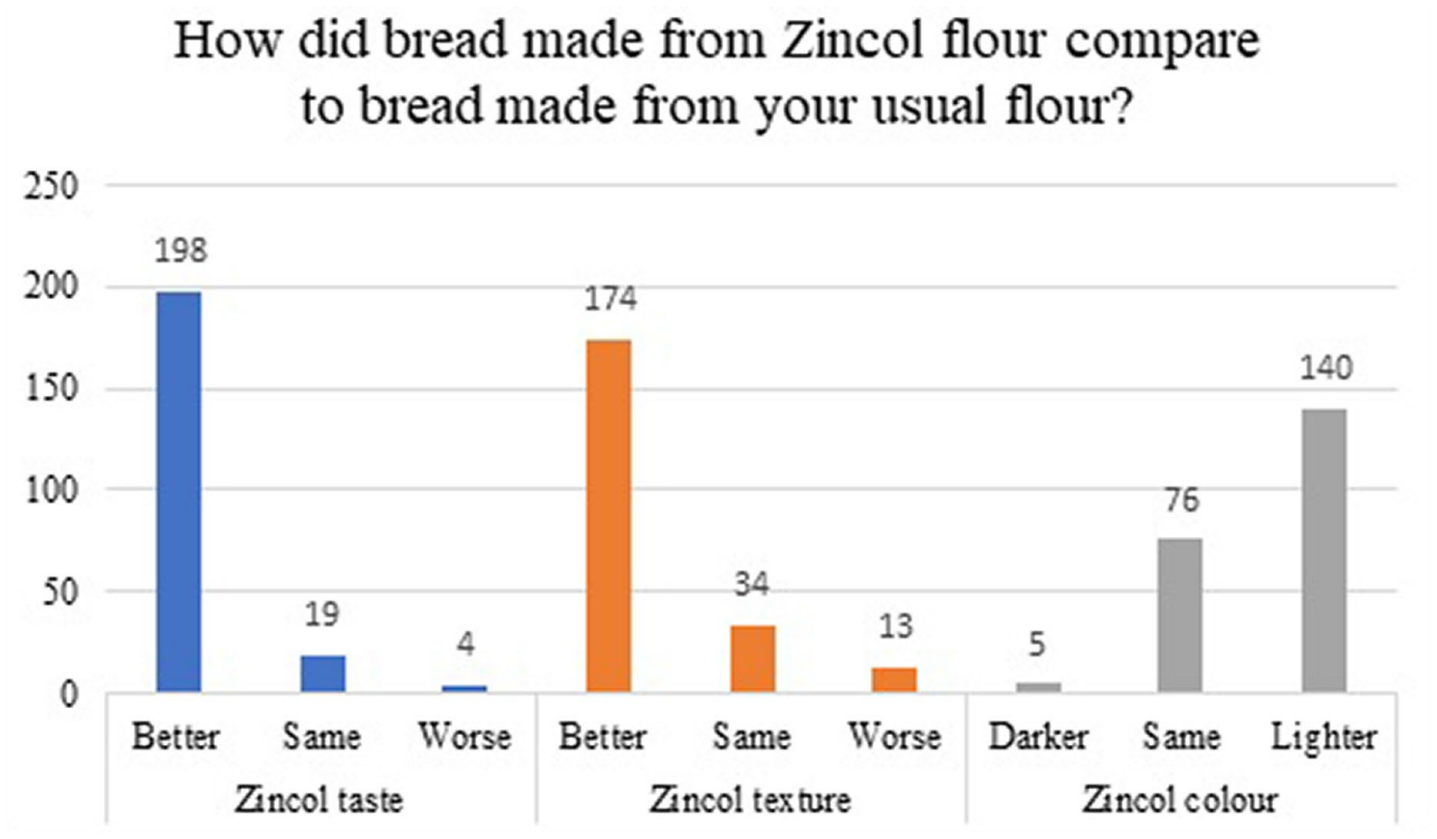
The participant responses to questions about Zincol-2016 bread quality compared to their usual variety.

Farmers who had indicated that they had kept some Zincol-2016 grain separate from their other variety post-harvest were asked how they used the retained grain. Of the 278 farmers who responded, the majority (79%) reported having more than one use for the grain. As shown in Figure 2 the most frequently given responses were that the grain had been used for consumption within their own household (*n* = 221), gifted or shared with neighbors (*n* = 144), or was stored for multiplication sowing in the following season (*n* = 227). Of those who consumed the Zincol-2016 grain within their own household, 29% had consumed it for less than 3 months (*n* = 64), 57% consumed it for 3–6 months (*n* = 127), and 14% consumed it for more than 6 months (*n* = 30). Of those who had consumed bread made with Zincol-2016 grain, most felt it had a better taste (*n* = 198, 90%), better texture (*n* = 174, 79%) and lighter color (*n* = 140, 63%) than bread made with their usual variety (Figure 3). Only a minority of farmers used the grain as payment to a landowner (*n* = 6) or as payment to laborers (*n* = 31) and 19% (*n* = 52) reported that they had sold the Zincol-2016 grain.

#### Pandemic effects

Farmers were asked if the COVID-19 pandemic impacted on the amount of grain they sold in 2020 to which 49% responded that they had sold the same amount as usual, 23% had sold less than usual, and 28% had sold more than usual.

### Focus group discussions with Peshawar farmers and millers

A total of 12 farmers and 12 millers agreed to take part on the FGDs. Information derived from the thematic analysis enabled the identification of two main themes: (1) Enablers for scaling up of biofortified wheat and (2) Challenges and considerations for scaling up.

### Enablers for scaling up of biofortified wheat

Among the enablers for scaling up we identified four subthemes: (1.1) Perceived health benefits; (1.2) Improved grain quality and production; (1.3) Willingness to produce and process biofortified wheat if provided with support; and (1.4) An opportunity for millers to expand their business.

### Perceived health benefits

The farmers and the millers who had produced and processed the biofortified wheat, recognized that adding minerals to the wheat or flour through biofortification or fortification was beneficial to alleviate mineral deficiencies among the population. The health benefits were seen as an incentive to produce and/or process biofortified wheat.

“Sir, the reason we consented (to grow biofortified wheat) was that there was deficiency in our country, children weakness, their mothers’ weakness, vitamin deficiency, when we read your description we found good things in it, and that wheat was beneficial for us. We consented for the purpose of having a healthy society” – A farmer.

### Improved grain quality and production

The farmers acknowledged that the process of growing biofortified wheat differed from their usual ways of farming (i.e., the way in which the land is prepared) and required additional care. However, farmers did not verbalize any concerns about the process of growing biofortified wheat or about the wheat grain provided to them during the trial. On the contrary, farmers expressed satisfaction with the quality of seeds and believed that the zinc sprays enabled the grain to grow stronger and be of better quality. Farmers also believed that the sprays were beneficial to the soil and felt that the chemicals they received increased their yield.

“It came up very good and its production was beautiful, we were very much happy with it. Every grain of wheat was very big and beautiful, every grain of it was like a red berry, it was the mercy of Allah and it happened due to the grace and mercy of Allah” – A farmer.

“It was very good because when we grew it the production was more than it used to be and additionally zinc and everything were given to it, so it brought a very good result” – A farmer.

The farmers expressed that they did not have any issues while growing Zincol-2016 for the BiZiFED trial, other than those that they would normally face while growing a non-biofortified variety. These common challenges included disease and weather issues.

“In the name of Allah, the most merciful, the most compassionate Mr. Rashid, when we would do farming previously, it would be on our (inaudible) but when you came, the method you did the wheat farming it was in a very good way, and we were to get its products but unfortunately some storms and rain occurred which caused the wheat’s grain to remain incomplete” – A farmer.

### Willingness to produce and process biofortified wheat if provided with support

The farmers and millers who produced and processed the Zincol-2016 wheat grain expressed that, given the benefits of biofortified wheat for the population, their production and business, they would be willing to produce and process it if given support (i.e., technical help, machinery, soil, fertilizers, seeds, wheat, and training) from the government or other organizations.

“Absolutely. We will be growing yeah (multiple voices). This is beneficial for us, this thing is good for our coming future, we like it very much, yes. We want the government to help us like that then God willing we will keep growing this. We request the government to help us the way you came last year and helped us in terms of chemicals, soils, wheat seeds” – A farmer.

“If the government provides us with biofortified wheat, then we are ready by all means to grind it. If we get this sort of wheat we will grind and sell in the bazaar” – A miller, Mill 1.

Some participants indicated the need for government initiatives to promote the scale up of biofortified wheat. A miller expressed his belief that improved community benefit would arise from a government supported program of biofortification.

“If the government supports this program as this work is done for the welfare and good of our people. Therefore, we should participate in it, and we should become part of it. If this done by the government then it would be carried out with enforcing people and it would develop easily. Instead of doing it privately if it is carried out through the government then it would bring up better results” – A miller, Mill 1.

The farmers expressed that receiving support during the BiZiFED2 trial in the form of soil, seeds, fertilizers and knowledge enabled farmers to increase their production and allowed unused lands to be re-used. Farmers expressed their interest for continuing to receive the support provided during the trial and expressed their concerns of a decline in production when the trial came to an end.

“The chemicals and the seeds increased our products and as Mr. (Name) told you that before when we would plough a field then we would plant a mound (37.324 kg) or two in it, but this time, Praise be to Allah, they showed us a proper limit and we used Urea as well in its proper limit, God willing. We were expecting after that as well, but it did not happen. God willing, we will fully cooperate with you if we receive all these things next time again” – A farmer.

### An opportunity for millers to expand their business

Discussions with millers also revealed that milling biofortified wheat was beneficial, not only for the potential health benefits that this could confer to the population, but as an opportunity to expand their business and possibly gain interest from the consumers, as had occurred when millers fortified wheat with support of the World Food Program (WFP).

“If we get it (biofortified wheat) then we would accept it with happiness, because we try to expand our business so it would help us to expand our business. And we would be able to provide people with such standard flour which would be good for their health, and it would meet their nutritional requirements as well” – A miller, Mill 1.

Participants from both mills believed that improving the nutritional value of the wheat through the WFP fortification initiative had boosted consumer demand, and one participant suggested that an identifier label or stamp may help consumers differentiate fortified products from standard varieties.

“Our production has increased with it (fortification) because the item that is put into it, which is iron, that is in fact for some requirement such as some illness and so. Therefore, people consume it with good interest” – A miller, Mill 2.

“Whichever market this flour goes, we have told all of our customers regularly that you should first look at its own fortified monogram and then buy the flour so that you make sure this thing is available in it” – A miller, Mill 1.

### Possible challenges and other considerations for scaling up biofortified wheat

Four sub-themes were identified as possible challenges and considerations for scaling up. These were: (1) Unfamiliarity with the biofortification process and crop; (2) Production costs; (3) Need of support from the landlord; (4) Millers’ beliefs about local wheat; and (5) External threats to supply chain of wheat and resources (i.e., COVID-19).

### Unfamiliarity with the biofortification process and crop

Prior to the BiZiFED2 trial, it appeared that neither farmers nor millers had previously produced or processed biofortified wheat, nor did they know what biofortification was. The farmers that produced biofortified wheat for the first time during the BiZiFED2 trial suggested to the research team that they use a simple local name to refer to biofortified wheat as they would find easier to understand.

“My suggestion is that you give us some advice, for this wheat which you call it biofortified, choose a name which a farmer finds that name sweet and dignified, yeah. Just call it ‘Sona’ [Sona is a fertilizer name used in this conversation but is an Urdu language term as well which is widely used in Pakistani Pashto, and it means ‘gold’ so in my judgement the famers here use it in this sense – Transcriber] wheat as a lot of hard work is done on it. As ‘Sona’ has a high quality in the soil so is this wheat” – A farmer.

Some millers described not being aware of what biofortified wheat was despite previously having ground it for the BiZiFED2 trial, but they were open to receiving more information about it. A miller believed that the farmers should be given training on the process since they would be the ones that grew the wheat.

“Those who need to be told first are the farmers as they are the one who grow the wheat and that wheat comes to us after that. We have not been briefed about this, so when they come then we will receive briefing about it. When they teach us, then good results will be achieved” – A miller, Mill 1.

The millers that had not ground Zincol-2016 before, expressed their beliefs that the quality of the local wheat was poor, hence they would not be interested in milling biofortified wheat if it was grown locally unless they had evidence that the quality of the flour produced by the biofortified grain was good.

“We will not be paying attention to this wheat firstly because its bread is weak, and secondly, we will see next year, if the wheat is good then we will be buying it. Although we do not buy the local wheat because even if we use it then it gets returned to us from every side, the wheat that particularly the wheat that is native to Peshawar” – A miller, Mill 2.

### Production costs

For the farmers, the costs of the chemicals required to support biofortified wheat crops was the main barrier to its adoption. Farmers expressed not being able to afford the chemicals as they needed to prioritize other expenditures due to severe resource limitations.

“Yeah, white fertilizer black fertilizer, chemicals, if these things are expensive then we will not be able to afford it, because we would be wondering whether to spend what we earn through labor work on the field or on ourselves. So, if we cannot do the crops, it would be due to the poverty and helplessness” – A farmer.

“Yes, it does require expenditure and we cannot afford it; it needs fertilizers, garbage [a particular garbage, mostly consisting of ashes which people use as natural fertilizer – Transcriber], and we cannot afford all of these. We can only put soil and so into it” – A farmer.

The price of the biofortified wheat was not directly mentioned by millers as a barrier to processing it, but they did state that when wheat prices increased, for example as they did during the COVID-19 pandemic due to supply chain issues, the price of their product also increased, and consumers may choose not to buy their flour. This suggests that millers may not be willing to mill biofortified wheat if the cost to consumers exceeded the cost of government-produced wheat.

“No, no, there is shortage of wheat. It is not available at all. When it is available then its price is 5,000 Rupees, yeah, when we flour it then people do not buy it because there is a big difference between the 5,000 and the price of the government wheat, the difference is almost 1,200 Rupees at this time” – A farmer.

### The need for support

Farmers and millers both described a requirement for third party support if they were to produce or process biofortified wheat. Decisions concerning the type of crops cultivated by the farmers was not only dependent on the farmers themselves but also on their landlords, which would ultimately impact on whether they would be able to grow biofortified wheat crops.

“We have (Name of landlord) land, we cultivate it. Whatever he orders us we cultivate the land in accordance to that” – A farmer.

Typically, farmers gave a proportion of their crop to their landlord and the rest was retained by the famers for self-consumption or for selling, either to millers or in the markets. Some of the money earned from the farmers’ sales was used to buy farming resources, but they also expressed a need for more support from their landlords for such expenses.

“There are a lot of expenses, the farmers spend money on tractor. The landlord just halves it [the harvest] and they do not care about anything else. There are expenses, at least half of those expenses should be accepted by the landlords” – A farmer.

Millers also believed that if there was any special equipment or training to process biofortified wheat they would need support from a third party.

“Whatever machinery is necessary for this, the government should provide us with, and we will go along with it” – A miller, Mill 1.

### External threats to the supply chain of wheat and resources

Market and road closures and transportation disruption were a threat to the wheat and resource supply chain (e.g., fertilizer and transport) and economy of the farmers and millers during the COVID-19 pandemic. Both groups described how the disruption in transportation would cause delays or an inability to deliver their products to the market or to their customers, making it difficult to sell their products.

“The disease that Corona [COVID-19] has brought has had a big impact. As my friend said when we take a crop to the bazaar, it is a challenge. Firstly, vehicles are not available and so going to the bazaar is difficult but when we manage to take it to the bazaar then there is the issue of selling; when we sell it, it goes for cheaper but when we buy something it cost expensively for us” – A farmer.

“Due to the Corona [COVID-19] the transportation has been affected very much. A work that would have been done for ten rupees before, has turned to be done for 100 rupees. Therefore, for a poor man wheat in the open market became very expensive. The wheat that the government would subsidize, and we would grind, that flour was available to the public with subsidy. But the private wheat’s transport cost doubled for the flour mills because of the closing of vehicles stations, and lack of transportation. So, this was a loss for the mills” – A Miller, Mill 1.

Farmers and millers also noted the impact of reduced opening times at the markets where they usually sold their products. To ensure that products were sold, farmers would directly sell their products to the mills or sell them at a lower price which represented losses for them. Both farmers and millers expressed that the market closures would cause shortages and therefore an increase in the price of the resources (e.g., fertilizers and wheat) required for producing their products.

“It would certainly have been affected because it is about the bazaar, when the bazaar is closed then it definitely gets affected. When a product does not reach the bazaar on time then the product that was supposed to be sold for ten, now it gets sold for eight. This issue is there yeah” – A Farmer.

However, these adverse circumstances may have been short-lived as participants in subsequent focus groups (conducted in February 2021) did not describe market closures and attributed this to support from the government. The millers acknowledged the work of the government in reducing the shortage of wheat during the pandemic and reducing its price.

“The price was high at that time because the wheat was short and wasn’t available. But at the present time the wheat is gradually increasing from every side and the government provides a lot of facilities, so a month ago our wheat was 1,250 Rupees and now it is 1,100 Rupees, and due to the government wheat, the flour is getting cheaper” – A miller, Mill 2.

The farmers expressed that the COVID-19 pandemic led to an unstable labor force as they were not able to leave their homes to go to work. This would affect their farming activities as those who had other jobs in addition to farming would not be able to afford the resources required to grow the wheat. The farmers explained that this instability in the labor force would cause economic difficulties among their consumers and hence their products would not get sold.

“It affects the work of a laborer as he cannot find work, so it impacts it in a big way. The farmers’ products do not get sold” – A farmer.

Millers, however, believed that people would continue to consume the same amount of flour during the pandemic therefore they would still be able to sell their products.

“Flour is something which is for daily consumption, therefore there has been no impact on it” – A miller, Mill 2.

## Discussion

Zinc biofortified wheat may be a sustainable strategy to increase the dietary zinc intake among populations with low access to nutritious diets (3). Given the importance of the producers’ acceptability of the biofortified crop to enable successful scaling up of biofortified wheat in Pakistan, the aim of this mixed methods study was to explore Pakistani farmers’ and millers’ experiences and attitudes towards the production and processing of zinc biofortified wheat.

The survey and focus group data provided evidence that the farmers were satisfied with the Zincol-2016 wheat variety. Almost half of farmers (47%) who received Zincol-2016 seed to grow in the 2019–2020 season, stored a portion of the grain harvest and expanded its production in the following season (2020–2021), which suggests a preference of Zincol-2016 over their existing varieties. In addition, in the focus groups the farmers expressed their willingness to grow the biofortified crop given the perceived health benefits of the grain. Our findings are similar to previous studies in Uganda and Nigeria that investigated stakeholders’ perceptions towards the adoption of biofortified crops, where farmers expressed a positive response to agronomic biofortification (14, 17), particularly if awareness of the benefits of biofortification was high (14).

In our study we were able to identify some potential motivators that could increase the likelihood of biofortified wheat adoption among producers and processors. These included perceptions related to the superiority of the grain yield and flour quality, perceived health benefits of the biofortified flour, acceptability of the flour among consumers, and potential for increased marketing opportunities. It was also noted that adoption is likely to be improved if support, in the form of training and resources, is provided alongside the biofortified seed.

Crop performance was the most cited motivators for growing Zincol-2016 among farmers, who observed that the quality of the Zincol-2016 seeds, together with the fertilizers, increased their yield. Two thirds of farmers who cultivated the Zincol-2016 grain in the following season (2020–2021) cited good growth, disease resistance and yield as reasons for why they chose to do so. Farmers who took part in the focus groups had no concerns about the health of their Zincol-2016 crops above and beyond the common challenges they regularly faced (i.e., common fungal disease and weather issues). These findings provide promising indicators for the success of scaling up of biofortified wheat, since maximizing yield and reducing production threats due to disease positively impact the farm income potential (26). Crop resilience to adverse climate conditions is a major factor affecting farming decisions. The frequency of extreme climate events is increasing in Pakistan as a consequence of climate change, and include extreme heat wave occurrences, a shortfall in irrigation water and drought conditions at sowing (18). This highlights the critical importance of developing new crop varieties that consider grain resistance, crop resilience and yield, alongside improved nutritional quality.

Production costs are a key factor that influence producers’ intention to produce biofortified crops, particularly in relation to the cost of foliar zinc fertilizer application, which may be in addition to soil enrichment with zinc and organic fertilizers. Foliar zinc fertilizers increase the zinc content and quality of the wheat crops and have been used with Zincol-2016 to enhance the grain’s performance (6, 19, 27). However, data suggests that the use of fertilizers in Pakistan is low (19). The survey with the farmers showed that the majority (83%) of participants reported that they did not apply foliar zinc fertilizer to their wheat crops, with more than half stating that their decision to apply foliar fertilizer would depend on the costs incurred. This was supported by views expressed in the focus groups, with farmers explaining that they would not be able to afford the fertilizers due to severe resource limitations. Wheat (input and output) prices in Pakistan are regulated by the federal government (28), and farmers are not incentivized for the commercial production of foods with higher nutritional qualities. Therefore, as it has been previously discussed (29), higher production costs may demotivate farmers to choose zinc biofortified wheat varieties or use zinc fertilizers if these are perceived as necessary to achieve the desired enhanced quality for market advantage.

A particularly striking finding of the farmer’s survey is that, with a starting provision of sufficient grain for half an acre of cultivation, seed saving, and multiplication saw this extend to nearly one third (31%) of the cultivated wheat area just 1 year later, for the 47% of initial farmers who continued to grow Zincol-2016. It also showed a strong preference of home consumption and gifting of seed to neighbors, rather than more commercial transfers to landowners, workers, or sale. Data from both focus groups and survey shows that seeds were retained to sow for the next season, providing evidence that an initial investment for the provision of seed could be a sustainable strategy to support the reduction of micronutrient deficiencies if the crop maintains its high zinc trait over successive growing season.

Compared to control flour, the additional zinc content of the Zincol-2016 flour produced in this study was 3.69 mg Zinc/kg (3). Farmers and millers who produced or processed Zincol-2016 recognized the importance of additional micronutrient content of the biofortified wheat to their community. Likewise, 40% of survey participants considered the nutritional qualities of the crop an important motivator when deciding whether to sow Zincol-2016 in the following season. A study conducted in Nigeria suggested that adoption of biofortified cassava among farmers may be improved by increasing their awareness of the health benefits of the crop (14). These findings, however, are in contrast to a Ugandan study which reported that farmers growing biofortified banana did not value the improved mineral content of the product since they believed that consumers were unconcerned about nutritional value and only cared about buying enough food to feed their families (30). Our earlier study on consumers perceptions of biofortified wheat challenges this view, as consumers of zinc biofortified flour valued the health benefits that they perceived it gave, which may suggest a preference over standard varieties (21).

The results of our study suggest that decisions to grow biofortified wheat does not only rely on the farmers, but also on the landowners who may have ultimate control of what the farmers grow. Moreover, it is possible that farmers may not want to invest in zinc fertilizers or improvements in the soil if their tenure is uncertain. A study in Pakistan (31) suggested that tenure influenced farmers’ decisions regarding soil and yield improvement measures, such as fertilizer application. Therefore, stakeholders wishing to promote adoption may need to engage landowners and landlords in discussion to actively influence their farm management decisions.

The present study was conducted during a critical period of the COVID-19 pandemic, when intermittent lockdowns were in place across Pakistan. In the FGDs farmers described their experiences during lockdown, including difficulties with access to markets affecting the price of fertilizers and pesticides and their ability to sell their products, and increased prices of basic food items. This concurs with similar reports from Pakistan (32) and India (33). A survey by Hussain et al., showed that farmers in Pakistan faced many difficulties with crop cultivation during the COVID-19 lockdown, including a perceived increase in price of fertilizers and pesticides and difficulties accessing markets affecting the price of foods (32). Similarly, a survey in India reported that farmers faced delays on their ability to sell their products due to travel restrictions, representing a financial loss for farmers who were unable to safely store their goods, and difficulties accessing a varied diet due to the lack of availability of certain foods or increased prices (33). These studies highlight the fragility of the food system and a need for strategies to support farmers in their ability to sustainably produce nutrient rich foods even under challenging circumstances. Although the COVID-19 pandemic is currently controlled, other external threats such as climate change and international conflict pose a potential risk to global wheat production and resulting food security and dietary diversity of vulnerable populations (18).

Evidence suggests that a key driver to the adoption of Zincol-2016 and other biofortified wheat varieties in rural areas in Pakistan is the provision of adequate support for both producers and processors of the grain. Our survey and focus group discussions suggested that most farmers were willing to grow biofortified wheat if given affordable access to foliar zinc fertilizers and training on their application. A previous study in Pakistan exploring the factors influencing the adoption of improved wheat varieties in rural areas revealed that access to extension services and micro credit schemes was found to be a key factor (34). In this context, the farm advisory/extension services visited the farmer, inspected crop health, and took soil samples (for onward lab recommendations) to issue advice. These services were provided free of cost to the farmer by the provincial agriculture department as well as by private fertilizer companies. These services provided an opportunity for creating awareness about the biofortified crops, promoting the adoption of biofortified varieties and provision of training for the farmers.

Finally, our study revealed that the millers considered consumer acceptability and the quality of the food staple when deciding whether to adopt new technologies. The millers who did not process Zincol-2016 grain in our study expressed that they would not adopt it until they had evidence that the flour and bread it produced were of good quality. Our survey data indicated that the majority of farmers who had consumed the Zincol-2016 flour believed that the bread it produced had a better taste and texture compared to their usual varieties. Similarly, our earlier study of consumers’ perceptions of Zincol-2016 (21) revealed that participants felt that the resulting flour had good sensory and cooking attributes (21), which suggests that good quality produce may be a facilitator for the adoption of Zincol-2016 among both consumers and producers. The importance of consumer acceptability was also highlighted in one study (35) where findings showed that millers were reluctant to invest in fortification spraying technologies to increase the nutritional quality of food staples as they perceived that the resulting produce (in this case micronutrient fortified rice) was not well received by consumers (31).

To our knowledge, this is the first study that has been conducted exploring producers’ experiences of growing biofortified wheat in Pakistan. The quantitative survey 418 of farmers, together with the qualitative exploration of experiences and attitudes allowed the triangulation of data and added richness and depth to the findings. This study also has some limitations. The farmers in the focus group discussions received support from the BiZiFED2 project, which included the provision of the seed, fertilizers, and the purchase of their crop at a favorable rate. The farmers in the survey received free Zincol-2016 seed the previous growing season and soil fertility data at the time of their selection, which could have introduced bias to their responses: they do not represent an unbiased sample of Punjab Province wheat growers in relation to biofortified wheat cultivation. Moreover, our survey did not collect the reasons for why some farmers did not grow Zincol-2016 in the following season. Additionally, the views of farmers not involved in the BiZiFED2 trial were not sought, and it is possible that their views may differ from those of farmers involved in the study.

In conclusion, our study suggests that biofortified wheat was well received among the producers in KP and Punjab provinces. Although both farmers and millers valued the nutritional qualities of the crop, farmers felt that crop performance and yield were among the most valued characteristics of the grain, while millers saw it as a marketing strategy and would be willing to adopt it due to the high quality of the product. Further awareness and training about the benefits of biofortification is required among both farmers and millers. Farmers require support to acquire and utilize zinc foliar fertilizers to optimize the zinc content of the Zincol-2016 grain. The results of this study strongly suggest that farmers and millers are willing to produce biofortified wheat providing support is given in the form of resources and training. These finding can inform the scaling up of biofortification for the provision of more nutritious foods to populations, particularly in areas outside the reach of centralized fortification interventions.

## Data availability statement

The raw data supporting the conclusions of this article will be made available by the authors, without undue reservation.

## Ethics statement

The studies involving humans were approved by Ethical approval granted by the University of Central Lancashire (REFTEMH1014), Khyber Medical College Peshawar (reference BZ/000628) and University of Nottingham (BIO-1819-001A) ethics committees. The studies were conducted in accordance with the local legislation and institutional requirements. The participants provided their written informed consent to participate in this study.

## Author contributions

NML, VHM, ELA, EJMJ, HO, MZa, MZi, and SA contributed to the design and development of the study. UM, HO, SA, ELA, MZi, and MZa managed data collection. MC-R and UM completed initial coding and analysis of the qualitative data, which were double checked by VHM and EJMJ. SA and ELA cleaned and analyzed the survey data. M-CR integrated the qualitative and quantitative data and wrote the manuscript. VHM, NML, and EJMJ revised the paper and finalized the manuscript. All authors contributed to the article and approved the submitted version.

## Abbreviations

KP: Khyber Pakhtunkhwa
BiZiFED2: Biofortified zinc flour to eliminate deficiency in Pakistan
WP: Work packages
