# Supplementary material for: Farmers’ and millers’ experiences and attitudes towards the production and processing of zinc biofortified wheat in Pakistan: a mixed methods study
Source: Front Nutr. 2023 Oct 24;10:1158156. doi: 10.3389/fnut.2023.1158156 (PMC10629276; doi:10.3389/fnut.2023.1158156)
Supplement: Supplementary file 1 [file Data_Sheet_1.PDF]

Contents

Supplementary Tables 2
Focus group discussion Topic Guides 9
Millers 9
Farmers 11

BiZiFED

Supplementary Tables

Table 1 List of questions asked to the participants

Quetion	Original survey question text	Shortened data field name for analysis	Entered data format	Pre-determined text options	Condition
	start	SurveyDate_ISO	date (YYYY-MM-DD)		all
	new column	SurveyDate_UK	date (DD/MM/YYYY)		all
	Farm GID	FarmGID	From project list		all
Q1	Farm typology	FarmTypology	From project list		all
Q2	How many acres of wheat did you grow last season (2019/20)?	AcresWheat_2019	Number		all
Q3	How many acres of Zincol wheat did you grow last season (2019/20)?	AcresWheatZincol_2019	Number		all
Q4	Did Covid-19 affect the amount of wheat grain you sold last season (2019/20)?	C19impact_WheatSales	Pre-determined text	Less than usual / more than usual / same as usual	all
	Section 1: Zincol Questions	*ZincolQuestions*	*No answer required*		
Q5	Did you keep any of the harvested Zincol grain separate, or did you mix it all with other varieties of wheat grain?	Zincol_SeparateOrMix	Pre-determined text	Kept some Zincol grain separate / Mixed it all with other varieties of wheat grain	all
Q6	What did you do with your Zincol wheat crop in 2020 (after harvest)?	Zincol_postHarvest	Pre-determined text	Text string of all following answers where answer = 1	If 'kept separate'
	Sold some or all	Zincol_SoldSomeAll	0 or 1	0 (no) or 1 (yes)	If 'kept separate'
	Used as payment to landowner	Zincol_LandownerPayment	0 or 1	0 (no) or 1 (yes)	If 'kept separate'

Quetion	Original survey question text	Shortened data field name for analysis	Entered data format	Pre-determined text options	Condition
	Used as payment to labourers	Zincol_LabourerPayment	0 or 1	0 (no) or 1 (yes)	If 'kept separate'
	Consumed within my own household	Zincol_Hhconsumed	0 or 1	0 (no) or 1 (yes)	If 'kept separate'
	Gifted to another household or shared some seed with neighbouring landlord	Zincol_SeedGiftedShared	0 or 1	0 (no) or 1 (yes)	If 'kept separate'
	Stored safely as seed for multiplication during 2020/21 season	Zincol_StoreToSow	0 or 1	0 (no) or 1 (yes)	If 'kept separate'
	Other	Zinco_other	0 or 1	0 (no) or 1 (yes)	If 'kept separate'
	If other was selected, please make note of the reasons here.	Zincol_otherText	free text		if 'other'
Q7	For how many months did your family consume Zincol flour?	Zincol_ConsumeFlourDuration	Pre-determined text	Less than 3 months / 3-6 months / Longer than 6 months (still consuming)	If 'kept separate'
Q8	How did bread made from Zincol flour compare to bread made from your usual flour?	Zincol_CompareFlour	No answer required		If 'kept separate'
	Taste	Zincol_Taste	Pre-determined text	Better / Worse / Same	If 'kept separate'
	Texture	Zincol_Texture	Pre-determined text	Better / Worse / Same	If 'kept separate'
	How did the colour of bread made from Zincol flour compare to bread made from your usual flour?	Zincol_Colour	Pre-determined text	Lighter / Same / Darker	If 'kept separate'

Quetion	Original survey question text	Shortened data field name for analysis	Entered data format	Pre-determined text options	Condition
	Was there any difference noted by the Zincol grain buyers due to its phenotypic varietal differences, such as grain size and its colour?	Zincol_GrainBuyerNotes	Pre-determined text	Yes / No	If 'kept separate'
	If yes, which difference was noted?	Zincol_GrainBuyerNotesText	free text		if buyer noted 'yes'
	Section 2: Questions about the current growing season (Oct 2020 – June 2021)	*CurrentCropQuestions*	*No answer required*		
Q9	Are you growing the Zincol biofortified variety again, this season?	Zincol2020_grow	Pre-determined text	Yes / No	all
Q10	Why did you decide to grow Zincol again? Please rate the importance of each of these factors.	Zincol2020_reasons	No answer required		
	Availability of seed (new variety)	Zincol2020_SeedAvail	Pre-determined text	Not important / important / very important	if 'yes' to 2020 sowing
	Cost of seed	Zincol2020_SeedCost	Pre-determined text	Not important / important / very important	if 'yes' to 2020 sowing
	Growth and disease resistance	Zincol2020_GrowResist	Pre-determined text	Not important / important / very important	if 'yes' to 2020 sowing
	Market demand	Zincol2020_MktDemand	Pre-determined text	Not important / important / very important	if 'yes' to 2020 sowing

Quetion	Original survey question text	Shortened data field name for analysis	Entered data format	Pre-determined text options	Condition
	Grain yield	Zincol2020_Yield	Pre-determined text	Not important / important / very important	if 'yes' to 2020 sowing
	Quality of the flour from previous harvest	Zincol2020_FlourQual	Pre-determined text	Not important / important / very important	if 'yes' to 2020 sowing
	Nutritional benefit	Zincol2020_Nutrition	Pre-determined text	Not important / important / very important	if 'yes' to 2020 sowing
	Other	Zincol2020_Other	Pre-determined text	Not important / important / very important	if 'yes' to 2020 sowing
	If other was selected, please make note of the reasons here.	Zincol2020_OtherTxt	free text		If 'other' is yes
Q11	How many acres of Zincol are you growing this season (2020/21)?	AcresWheatZincol_2020	Number		if 'yes' to 2020 sowing
Q12	How many acres of wheat are you growing this season (2020/21)?	AcresWheat_2020	Number		if 'yes' to 2020 sowing
Q13	Did you save any Zincol seed from the last harvest to sow this season (2020/21)?	Zincol2020_savedSeed	Pre-determined text	Yes / No	if 'yes' to 2020 sowing
	Did you share any saved Zincol seed with family or neighbours, for them to sow this season (2020/21)?	Zincol2020_sharedSeed	Pre-determined text	Yes / No	if 'yes' to 2020 sowing
	Did you purchase new Zincol seed for this growing season (2020/21)?	Zincol2020_boughtSeed	Pre-determined text	Yes / No	if 'yes' to 2020 sowing

Quetion	Original survey question text	Shortened data field name for analysis	Entered data format	Pre-determined text options	Condition
Q14	Have you heard of the recently introduced biofortified wheat variety, Akbar-19?	Akbar19_var	Pre-determined text	Yes / No	all
	Are you growing Akbar-19 wheat this growing season (2020/21)?	Akbar19_growing	Pre-determined text	Yes / No	if yes to Akbar19
Q15	How many acres of Akbar-19 wheat are you growing this season (2020/21)	AcresWheatAkbar_2020	Number		if yes to Akbar19
	Section 3: Questions on fertilizer use and support required	*FertliserQuestions*	*No answer required*		
Q16	Did you know that Zincol also requires foliar application of zinc fertilizer (in addition to soil application) if you want to have maximum zinc concentration in the grain?	ZincolCult_KnowFoliarFert	Pre-determined text	Yes / No	if 'yes' to 2020 sowing
Q17	What factors might influence your decision about whether to apply zinc fertilizers?	ZincFert_factors	Pre-determined text	Text string of all following answers where answer = 1	if 'yes' to 2020 sowing
	What factors might influence your decision about whether to apply zinc fertilizers?/Availability of zinc fertilizers	ZincFert_avail	0 or 1	0 (no) or 1 (yes)	if 'yes' to 2020 sowing
	What factors might influence your decision about whether to apply zinc fertilizers?/Cost of zinc fertilizers	ZincFert_cost	0 or 1	0 (no) or 1 (yes)	if 'yes' to 2020 sowing
	What factors might influence your decision about whether to apply zinc fertilizers?/Access to credit	ZincFert_credit	0 or 1	0 (no) or 1 (yes)	if 'yes' to 2020 sowing

Quetion	Original survey question text	Shortened data field name for analysis	Entered data format	Pre-determined text options	Condition
	What factors might influence your decision about whether to apply zinc fertilizers?/Lack of knowledge about how to apply zinc fertilizers	ZincFert_knowledge	0 or 1	0 (no) or 1 (yes)	if 'yes' to 2020 sowing
	What factors might influence your decision about whether to apply zinc fertilizers?/Discolouration of leaves	ZincFert_leafcolour	0 or 1	0 (no) or 1 (yes)	if 'yes' to 2020 sowing
	What factors might influence your decision about whether to apply zinc fertilizers?/Ability to use it on other crops like rice (followed by wheat)	ZincFert_AltCropUse	0 or 1	0 (no) or 1 (yes)	if 'yes' to 2020 sowing
	What factors might influence your decision about whether to apply zinc fertilizers?/Preference for organic methods	ZincFert_PrefOrgCult	0 or 1	0 (no) or 1 (yes)	if 'yes' to 2020 sowing
	What factors might influence your decision about whether to apply zinc fertilizers?/Market demand/buyers interest	ZincFert_MktDemand	0 or 1	0 (no) or 1 (yes)	if 'yes' to 2020 sowing
Q18	Would you be more likely to apply foliar zinc onto wheat field being grown specifically for your own consumption?	ZincFert_HHconsumption	Pre-determined text	Yes / No	if 'yes' to 2020 sowing
Q19	Would you like to receive further training or information about the use of zinc fertilizer?	ZincFert_LikeTraining	Pre-determined text	Yes / No	all
	_index	_index	Auto-number	Entry by kobo software; Unique	all

Quetion	Original survey question text	Shortened data field name for analysis	Entered data format	Pre-determined text options	Condition
				record identifier.	

Focus group discussion Topic Guides

Millers

Welcome participants.

Introduce yourself and the FGD observer.

This focus group is part of the BIZIFED2 project, which involves partners from the Abaseen Foundation, Khyber Medical University and the University of Central Lancashire in the UK.

The purpose of this focus group discussion is to find out about local milling practices and any challenges you may have faced during the COVID-19 pandemic.

We are also interested to hear your views on zinc wheat. This is a new variety of wheat available in Pakistan, which has been bred for higher nutritional content. We will ask you some questions about that later.

So, to start with…

1. Please could you tell us what kind of mills you operate?

   Details such as type of mill, size of mill, types of grain milled etc.

2. Has the COVID-19 pandemic has affected your milling activities?

   If so, how?
   Were there any additional restrictions on flour mills?
   If yes, how did the additional restrictions affect procurement of grain or sale of flour?

3. Do you think the pandemic will influence your milling activities in the coming months?

   What safety procedures have you put in place for workers and customers?

4. In a normal year (before the pandemic), who are your main customers?

   For example, are they farmers or local householders?

5. Do you think the pandemic has affected the wheat market?

   Did the local markets stay open or were they closed?
   Did you sell your flour through different routes?
   Have you achieved the same prices for your flour?

6. Does your unit take part in Governments efforts to fortify wheat flour?

   If yes, do you know which mineral elements are fortified?

   Was the fortification programme disrupted during the pandemic? If so, how?

7. What do you understand by the term zinc wheat?

   Do you know how this new variety of wheat differs from standard varieties?

8. Have you ever been asked to mill zinc wheat?

   If so, was it for the BIZIFED2 project? If the answer is no, move onto question 10.

9. Did you notice any differences compared to other local varieties of wheat?

   Different aspects of performance e.g. ease of milling, quality of flour.

10. If zinc wheat becomes more widely used in this area, what would you need to consider in terms of milling this new variety?

    How would you prevent contamination with other varieties of grain and flour?

11. What kind of support would be useful for local millers in relation to zinc wheat?

    Do you need more information or resources?

12. Do you have any further questions or concerns about zinc wheat?

    Say that you will find out the answers and let them know.

13. Is there anything else you would like to say before we finish?

    Thank you taking time to participate in this discussion.

Farmers

Welcome participants.

Introduce yourself and the FGD observer.

This focus group is part of the BIZIFED2 project, which involves partners from the Abaseen Foundation, Khyber Medical University and the University of Central Lancashire in the UK.

The purpose of this focus group discussion is to find out about local farming practices and any challenges you may have faced during the COVID-19 pandemic.

We are also interested to hear your views on zinc wheat. This is a new variety of wheat available in Pakistan, which has been bred for higher nutritional content. We will ask you some questions about that later.

So, to start with…

14. Please could you tell us about farming practices here in Nahaqi?

    Details such as farm size, tenancy arrangements, range of crops etc.

15. Has the COVID-19 pandemic has affected your farming activities?

    If so, how?
    Were there any additional restrictions on farmers?
    If yes, how did the additional restrictions affect transporting or selling wheat?

16. Do you think the pandemic will influence your farming activities in the coming months?

    Can you access farm inputs such as labor, seed, fertilizer, manure and machinery?
    Would it influence your crop choice or any other factors?

17. In a normal year (before the pandemic), who do sell your wheat crop to, and what proportion of it do you sell?

    Do you retain some of the crop for household consumption?
    Was more of the crop retained for home consumption this year?

18. Do you think the pandemic has affected the wheat market?

    Did the local markets stay open or were they closed?
    Did you sell your wheat through different routes?
    Have you achieved the same prices for your wheat?

19. What do you understand by the term zinc wheat?

Do you know how this new variety of wheat differs from standard varieties?

20. Is this the first time you have grown zinc wheat?

    Why did you agree to grow it for this research project?

21. How did the zinc wheat perform compared to standard varieties of wheat?

    Different aspects of crop performance e.g. germination, emergence, crop vigour, growth, tillering, disease resistance, pest resistance.

22. Did you apply zinc fertilizers to the zinc wheat?

    If yes, at what stages of growth? Did it seem to be effective?
    If no, why not?

23. Would you consider growing zinc wheat next season?

    What factors might influence your decision?
    Do you think COVID-19 may influence your decision?

24. What kind of support would be useful for local farmers growing zinc wheat?

    Do you need more information or resources?

25. Do you have any further questions or concerns about zinc wheat?

    Say that you will find out the answers and let them know.

26. Is there anything else you would like to say before we finish?

    Thank you taking time to participate in this discussion.
